# COVID-19 Presenting With Thalamic Hemorrhage Unmasking Moyamoya Angiopathy

**DOI:** 10.1017/cjn.2020.117

**Published:** 2020-06-04

**Authors:** Ritwik Ghosh, Souvik Dubey, Biman Kanti Ray, Subhankar Chatterjee, Julián Benito-León

**Affiliations:** Department of General Medicine, Burdwan Medical College & Hospital, Burdwan, West Bengal, India; Department of Neuromedicine, Bangur Institute of Neurosciences, Kolkata, West Bengal, India; Department of General Medicine, Rajendra Institute of Medical Sciences, Ranchi, Jharkhand, India; Department of Neurology, University Hospital “12 de Octubre”, Madrid, Spain; Centro de Investigación Biomédica en Red sobre Enfermedades Neurodegenerativas (CIBERNED), Madrid, Spain; Department of Medicine, Universidad Complutense, Madrid, Spain

**Keywords:** COVID-19, SARS-CoV2, Moyamoya angiopathy, Thalamus

Neurological manifestations of the ongoing pandemic of coronavirus disease 2019 (COVID-19) have drawn significant attention in recent times, including a high incidence of acute strokes.^1^ However, stroke associated with moyamoya angiopathy has not been reported with new severe acute respiratory syndrome coronavirus (SARS-CoV-2) infection. We report a patient with SARS-CoV-2 infection presenting with thalamic hemorrhage and acute cognitive impairment unmasking moyamoya angiopathy.

**Figure 1:**
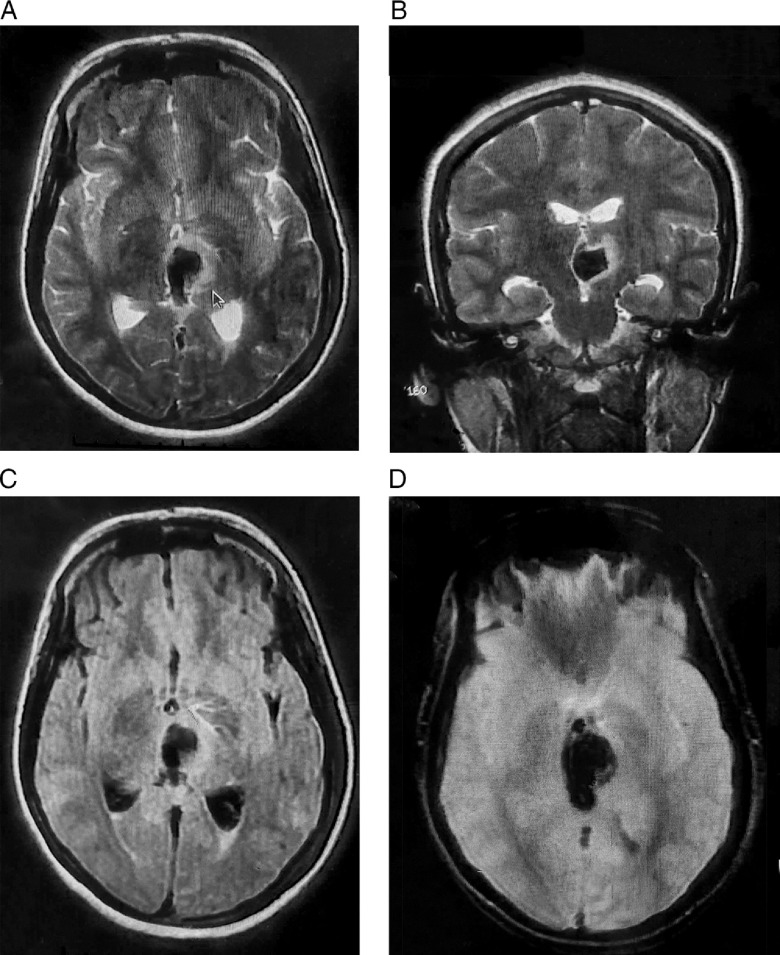
Contrast-enhanced magnetic resonance imaging of brain revealing non-enhancing focal altered intensity, which is hypointense in axial-T2 (A), coronal-T2 (B), and axial-fluid-attenuated inversion recovery (C) with signal blooming in axial-gradient echo sequences (D) at left thalamic area with perilesional edema and mass effect over third ventricle and associated intraventricular extension, suggestive of acute left thalamic intracerebral hemorrhage.

**Figure 2:**
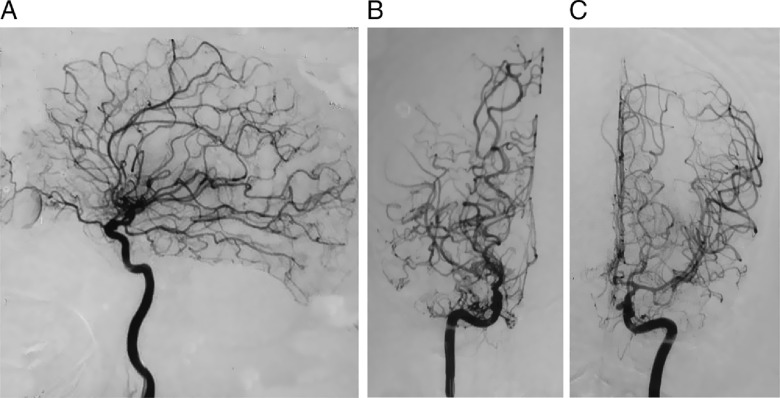
Digital subtraction angiography. Lateral projection displaying a moderately stenosed supra-clinoid left internal carotid artery (A), with well-developed extensive dense collaterals suggestive of moyamoya angiopathy. Antero-posterior projections displaying moderately stenosed supra-clinoid right (B) and left (C) internal carotid arteries, including M1 and A1 segments, along with well-developed extensive dense collaterals suggestive of moyamoya angiopathy.

A 19-year-old right-handed female college student with 14 years of continuous formal education and unremarkable medical history presented to the emergency department with acute onset of severe headache and decreased level of consciousness. She had a history of recent travel from COVID-19 endemic zone. According to her family members, she had a throbbing headache, fever, malaise, and decreased taste sensation since last 3 days. She was admitted with strict isolation protocols, and her airway, breathing, and circulation were secured. The patient’s nasopharyngeal swab test for SARS-CoV-2 by qualitative real-time reverse-transcriptase polymerase chain reaction assay was positive. A brief clinical examination was notable only for Glasgow coma scale of 12/15, conjugate eye deviation towards right side with a vertical skew and mute planter response. The next day after regaining full awareness, detailed neurological examination revealed severe impairment of recent memory (predominantly verbal memory was affected, whereas visual memory was relatively preserved) and new learning, oratorical confabulations, disrupted insight, impaired recognition, and erroneous recall order. She went on having repeated queries (perseveration) and lost track of recent conversations. She also had personality changes in the form of apathy and lack of self-motivation. This was associated with acalculia without language dysfunction, apraxia, alexia, agraphia, executive dysfunction, and neglect. Apart from these cognitive difficulties, she had right upper arm dystonic posturing and clumsiness without any other abnormalities in sensory, motor, cerebellar, and autonomic functions. Complete metabolic panel revealed no abnormality. A contrast enhanced magnetic resonance imaging of brain showed an acute left thalamic intracerebral hemorrhage (Figure 1), meanwhile a digital subtraction angiography revealed features of moyamoya angiopathy (Figure 2). Other directed investigations to establish etiology of moyamoya angiopathy were futile, and the diagnosis of moyamoya disease associated with COVID-19 was finalized. Patient was managed conservatively and discharged after 2 weeks with a plan for performing revascularization. Repeat cognitive assessment at 1-month post-stroke displayed persistent amnesia and confabulation with lesser severity and dystonia improved.

Impairments of multiple domains of cognition and emotional dysregulation may occur after thalamic stroke.^2^ The hippocampus and entorhinal cortex are connected to mammillary bodies, which in turn are annexed to the anterior nucleus of thalamus by mammillo-thalamic tract.^2^ This tract conjointly with fornix affix the anterior nucleus of thalamus into a network serving the learning and memory functions.^2^ Another pathway, the ventral amygdalofugal tract, aids to memory and emotional regulation, connecting amygdala with medial part of dorsomedial nucleus of thalamus.^2^ Behavioral patterns can be delineated on the basis of the main arterial thalamic territories.^3^ Paramedian strokes are associated with disinhibition syndromes, with personality changes, loss of self-activation, amnesia, and, in the case of extensive lesions, “thalamic dementia,” whereas anterior strokes generate mainly of perseverations and superimposition of unrelated information, apathy, and amnesia, as depicted in our patient.^3^

Cortico-fugal fibers fasten the motor and premotor areas to nucleus of Darkschewitsch and interstitial nucleus of Cajal (midbrain), controlling the vertical gaze.^4^ Downgaze is controlled by fibers to the rostral nucleus of medial longitudinal fasciculus (tectal area).^4^ Both of these pathways get disrupted by a lesion in medial thalamus giving rise to aberrant oculomotor changes as in our patient.^4^ Furthermore, damage in the centromedian or ventral intermediate nuclei, outside pallido-nigral territory can lead to disruption of the cortico–striato–pallido–thalamo–cortical loop resulting in dystonia.

Adult patients with moyamoya angiopathy usually present with intracerebral hemorrhage.^5^ Long-standing compensatory abnormal dilatation of lenticulostriate, chroidal, and thalamostriate arteries is cause of development of fragile moyamoya vessels.^5^ Two probable mechanisms for intracerebral hemorrhage are (a) development of microaneurysms in the periventricular moyamoya collaterals and (b) perpetual hemodynamic stress on the poorly developed collaterals, which make them easy prey for rupture and bleed.^5^

Many factors have potential to precipitate symptoms of moyamoya angiopathy. Commonly noticed are dehydration, systemic hypotension, recent infection, hot bath, vigorous exercise, emotional outburst, increased body temperature, crying, singing, blowing instruments, thyrotoxicosis and ketoacidosis and final common road being hyperventilation-induced cerebral hypoperfusion.^6^ Similarly, any systemic infection, such as COVID-19, could precipitate moyamoya angiopathy. Albeit, the exact pathogenesis is yet to be elucidated, potential mechanisms might be (a) increased cardio-embolic events and artery-to-artery embolism in the presence of infectious process and (b) hypercoagulable state in COVID-19.

Any sort of vascular inflammation is anticipated to result in hyperplasia and pathological remodeling of vascular smooth muscle cells, sprout of endothelial cells leading to angiogenesis that herald luminal narrowing and collateral formation.^7^ Increase in inflammatory cytokines as occurs in SARS-CoV-2-induced “cytokine storm” may influence the ring finger protein 213^8^ and caveolin-1 leading to moyamoya angiopathy.^9^

To conclude, SARS-CoV-2 infection and associated hemodynamic stress, emotional disruption, and cytokine storm could act as precipitating factors for moyamoya angiopathy. Thalamic stroke can present with acute cognitive impairment with behavioral changes impersonating acute psychiatric illness and that is why should be evaluated with utmost expertise.
